# Quantification and visualization of *cis*-regulatory dynamics in single-cell multi-omics data with TREASMO

**DOI:** 10.1093/nargab/lqae007

**Published:** 2024-02-02

**Authors:** Chaozhong Liu, Linhua Wang, Zhandong Liu

**Affiliations:** Graduate Program in Quantitative and Computational Biosciences, Baylor College of Medicine, Houston, TX 77030, USA; Graduate Program in Quantitative and Computational Biosciences, Baylor College of Medicine, Houston, TX 77030, USA; Jan and Dan Duncan Neurological Research Institute at Texas Children's Hospital, Houston, TX 77030, USA; Department of Pediatrics, Baylor College of Medicine, Houston,TX 77030, USA

## Abstract

Recent advances in single-cell multi-omics technologies have provided unprecedented insights into regulatory processes. We introduce TREASMO, a versatile Python package designed to quantify and visualize transcriptional regulatory dynamics in single-cell multi-omics datasets. TREASMO has four modules, spanning data preparation, correlation quantification, downstream analysis and visualization, enabling comprehensive dataset exploration. By introducing a novel single-cell gene–peak correlation strength index, TREASMO facilitates accurate identification of regulatory changes at single-cell resolution. Validation on a hematopoietic stem and progenitor cell dataset showcases TREASMO’s capacity in quantifying the gene–peak correlation strength at the single-cell level, identifying regulatory markers and discovering temporal regulatory patterns along the trajectory.

## Introduction

Recent advances in the field of single-cell genomics have enabled the direct link between chromatin accessibility and the transcriptome. Sequencing techniques such as SNARE-seq (1), SHARE-seq (2) and 10X Multiome can profile the two modalities simultaneously on the same batch of cells. In parallel, algorithms such as GLUE (3) and Seurat (4) can integrate single-cell RNA-seq and ATAC-seq datasets, aligning the two modalities for comprehensive analysis. However, despite these remarkable achievements, harnessing the full potential of multi-omics data for downstream analyses, particularly in the context of transcription regulation dynamics, remains a challenge.

ArchR (5), a widely used scATAC-seq analysis tool, employs Pearson correlation (6) to infer the links between scATAC-seq and scRNA-seq data. This approach involves clustering cells first, followed by computing the cluster-specific Pearson's correlation value. We propose that this method unfortunately oversimplifies the complex and continuous regulation processes that govern the interactions between chromatin accessibility and gene expression. For example, during stem cell differentiation, the correlation between *cis-*regulatory elements and gene expression gradually increases at various time points, representing a continuous and dynamic process. By reducing this intricate relationship to a single value, such as Pearson's *r*, we risk losing critical information and sacrificing the advantages of single-cell resolution. Moreover, such an approach overlooks cellular heterogeneity and fails to capture the regulatory dynamics within each cell cluster.

In light of these limitations, we introduce a statistical approach (7) to quantitatively infer the correlation between genes and opening regions in individual single cells. Leveraging this newly devised single-cell correlation strength index, we introduce TREASMO—an innovative software designed for Transcription Regulation Analysis in Single-cell Multi-Omics data. TREASMO features the unique single-cell correlation index, together with a wide array of user-friendly functions facilitating data preparation, statistical analysis for dynamic regulation and intuitive visualization.

In this manuscript, we introduce TREASMO and the comprehensive analysis pipeline with the 10X Multiome hematopoietic stem and progenitor cells (HSPCs) dataset as a use case. To facilitate a deeper understanding of our methodology, we provide Supplementary data containing detailed information on methods and implementation. The Python package is available at PyPI and open-source at GitHub (https://github.com/ChaozhongLiu/TREASMO) with detailed tutorials and example data.

## Materials and methods

### Software overview

**Figure 1.**
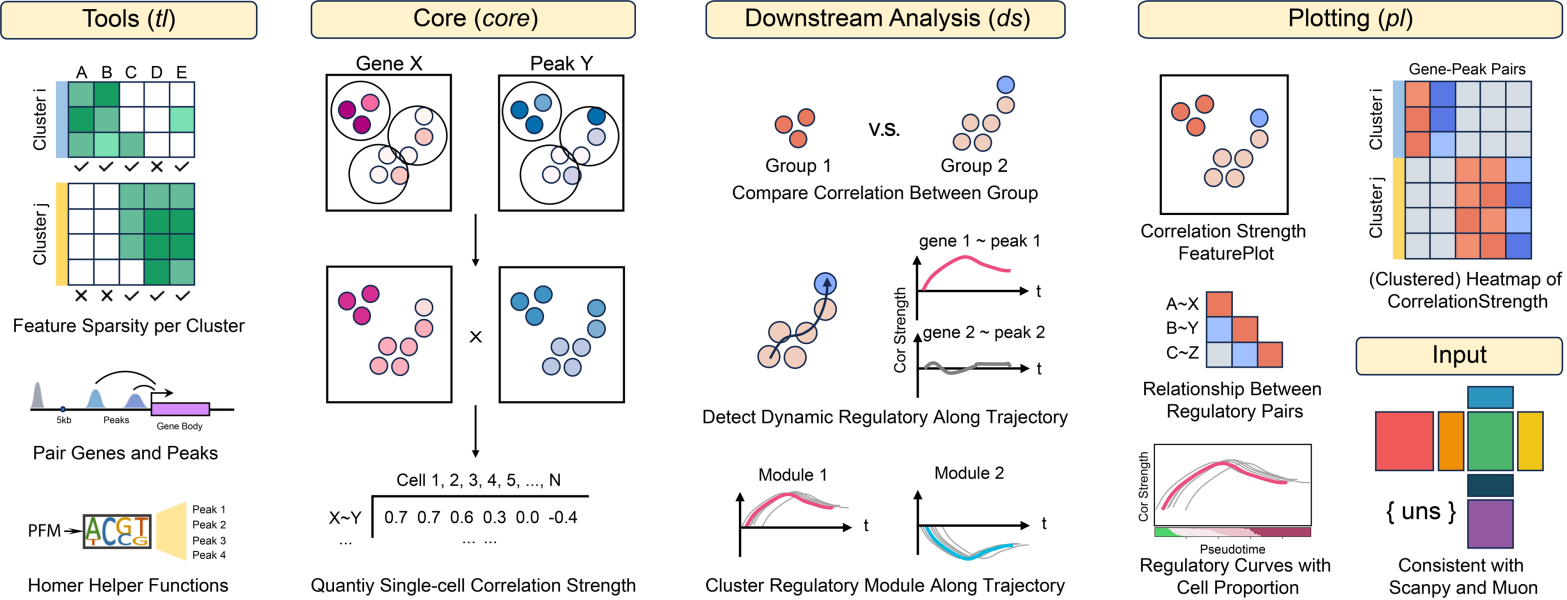
Overview of the four modules of TREASMO. TREASMO has four distinct modules, namely *tl*, *core*, *ds* and *pl*. The *tl* module provides useful functions to prepare the data; the *core* module quantifies the single-cell level gene–peak correlation strength; the *ds* module offers several functions to select regulatory pairs of interest, including comparing between groups/conditions, detecting dynamic pairs along the trajectory and clustering pairs according to the temporal patterns; and the *pl* module visualizes the results in various ways. The basic input format of TREASMO is AnnData and MuData, enabling seamless connection with Scanpy and Muon.

TREASMO, implemented as a Python package, adheres to the same data structure and design framework as Scanpy (8) and Muon (9), ensuring seamless transferability among these powerful tools. Comprising four distinct modules, TREASMO offers a comprehensive toolkit for single-cell multi-omics analysis (Figure 1): (i) the ‘tools’ (*tl*) module facilitates data preparation; (ii) the ‘core functions’ (*core*) module quantifies the gene–peak correlation strength at the single-cell level; (iii) the ‘downstream analysis functions’ (*ds*) module detects transcription regulation dynamics among groups, between conditions or along trajectories; and (iv) the ‘plotting functions’ (*pl*) module visually presents the results at various stages of analysis.

### Neighborhood-inferred single-cell correlation strength index

The correlation strength $L_{x,y}^{( i )}$ of cell $i \in \{ {1,\ 2, \ldots ,n} \}$ between gene ${{\bf x}}\ \in \ {\mathbb{R}}^n$ and peak ${{\bf y}}\ \in \ {\mathbb{R}}^n$ is defined as:


\begin{eqnarray*}L_{x,y}^{\left( i \right)} = \ \frac{{n \cdot \left( {\widetilde {{x}_i} - \bar{x}} \right)\left( {\widetilde {{y}_i} - \bar{y}} \right)}}{{\sqrt {\mathop \sum \nolimits_i {{\left( {{x}_i - \bar{x}} \right)}}^2} \ \sqrt {\mathop \sum \nolimits_i {{\left( {{y}_i - \bar{y}} \right)}}^2} }},\end{eqnarray*}

where *n* is the number of cells, $\bar{x}$ and $\bar{y}$ are the numeric mean values of ${{\bf x}}$ and ${{\bf y}}$, and ${{\bf \tilde{x}}}$ and ${{\bf \tilde{y}}}$ are the spatial lag values which are composed of weighted averages of cell neighbors. The spatial lag of a certain gene expression value in cell *i* is defined as:


\begin{eqnarray*}\widetilde {{x}_i} = \ \mathop \sum \limits_j {w}_{ij} \cdot {x}_{j\ },\end{eqnarray*}

where $j$ is the index of connected cell with $i$ in the neighborhood graph, and ${w}_{ij}$ is their connectivity weight. In single-cell multi-omics cases, we take the weighted nearest neighbor connectivity matrix, and fill in the diagonal with a value of 1.0, followed by a row standardization to derive the final ${{\bf W}}\ \in \ {\mathbb{R}}^{{\boldsymbol{n}} \times {\boldsymbol{n}}}$.

To speed up the computation and allow scalability, we used a vectorized implementation. Suppose ${{\mathrm{Z}}}_{\mathrm{X}}$ and ${{\mathrm{Z}}}_{\mathrm{Y}}$ are the z-scored forms of gene expression matrix ${{\bf X}} \in {\mathbb{R}}^{{\boldsymbol{n}} \times {\boldsymbol{p}}}$ and the ordered corresponding chromatin accessibility matrix ${{\bf Y}} \in {\mathbb{R}}^{{\boldsymbol{n}} \times {\boldsymbol{p}}}$, the correlation strength matrix ${{\bf L}} \in {\mathbb{R}}^{{\boldsymbol{n}} \times {\boldsymbol{p}}}$ between ${\mathrm{p}}$ gene–peak pairs can be calculated with:


\begin{eqnarray*}{{\bf L}}\ = {\left( {{{{\bf Z}}}_{{\bf Y}}^{{\bf T}}{{{\bf W}}}^{{\bf T}}} \right)}^{{\bf T}} \circ \left( {{{\bf W}}{{{\bf Z}}}_{{\bf X}}} \right) = \ \left( {{{\bf W}}{{{\bf Z}}}_{{\bf Y}}} \right) \circ \left( {{{\bf W}}{{{\bf Z}}}_{{\bf X}}} \right)\end{eqnarray*}

A global correlation is computed as the average of correlation strength for all the cells within the data to represent the average correlation between gene ${{\bf x}}$ and peak ${{\bf y}}$, which is a similar metric to Pearson's *r*.

### Processing of the 10X Multiome HSPC datasets

To showcase TREASMO’s capabilities, we applied it to investigate the 10X Multiome CD34 + HSPCs dataset (Supplementary Figure S1), unveiling the step-by-step analysis process and highlighting intriguing cases of transcription regulation dynamics discovered through this innovative approach.

We obtained the pre-processed and annotated dataset from https://www.kaggle.com/competitions/open-problems-multimodal/data. To manage computational resources, we randomly down-sampled the dataset into 15 000 cells, subsequently removing a minority of B-cell progenitor cells, resulting in 14 927 cells for analysis. Principal component analysis (PCA) was performed on the library size-normalized and log1p-transformed scRNA-seq data, while latent semantic indexing (LSI) was applied to the TF-IDF-transformed scATAC-seq data. Nearest neighbor graphs were constructed separately for each modality using Scanpy and Muon.

We then constructed a weighted nearest neighbor (WNN) graph by combining scRNA-seq and scATAC-seq data with Muon. Clustering was performed using the Louvain algorithm with resolutions set at 0.75 and 1.0. The final differentiation stage annotation was manually combined with the original clustering, which was then provided to Scanpy for partition-based graph abstraction (PAGA) and geodesic distance inference for trajectory and pseudo-time results. Embedding of all cells was generated by Scanpy force-directed graph drawing with the PAGA graph as initialization. Detailed processing information and metadata for the 14 927 cells can be found in our GitHub repository.

## Results

TREASMO receives as input the pre-processed single-cell multi-omics *MuData* object, prepared using the standard pipelines of Scanpy and Muon. It requires a neighborhood graph (saved as ‘*connectivities*’ in *MuData.uns*) for inferring single-cell correlation strength, clustering annotation and trajectory for downstream analysis. The choice of the clustering and trajectory analysis method does not influence TREASMO’s analysis. To ensure the selection of regulation dynamics gene–peak pairs of high quality, the *tl* module initially computes the per-group feature sparsity, a critical quality metric. Subsequently, the *tl* module efficiently pairs genes and peaks. This rapid function can either annotate genes with peaks within a specified genome range, or annotate transcription factors with binding sites using a reference BED file. The pairing information is then stored as a Pandas DataFrame with extra information columns, which serves as input for computing the correlation strength index between gene–peak pairs in the *core* module.

With either user-defined or *tl-*generated gene–peak pairs, the *core* module computes all gene–peak pair correlation strengths for each cell (see the Materials and methods). This index leverages neighborhood information to infer the correlation, representing the gene–peak correlation strength in a specific cell. Intuitively, this strength index provides valuable insights into each cell's contribution to the overall correlation within a cell population, allowing us to quantify the heterogeneous and continuous regulation process.

**Figure 2.**
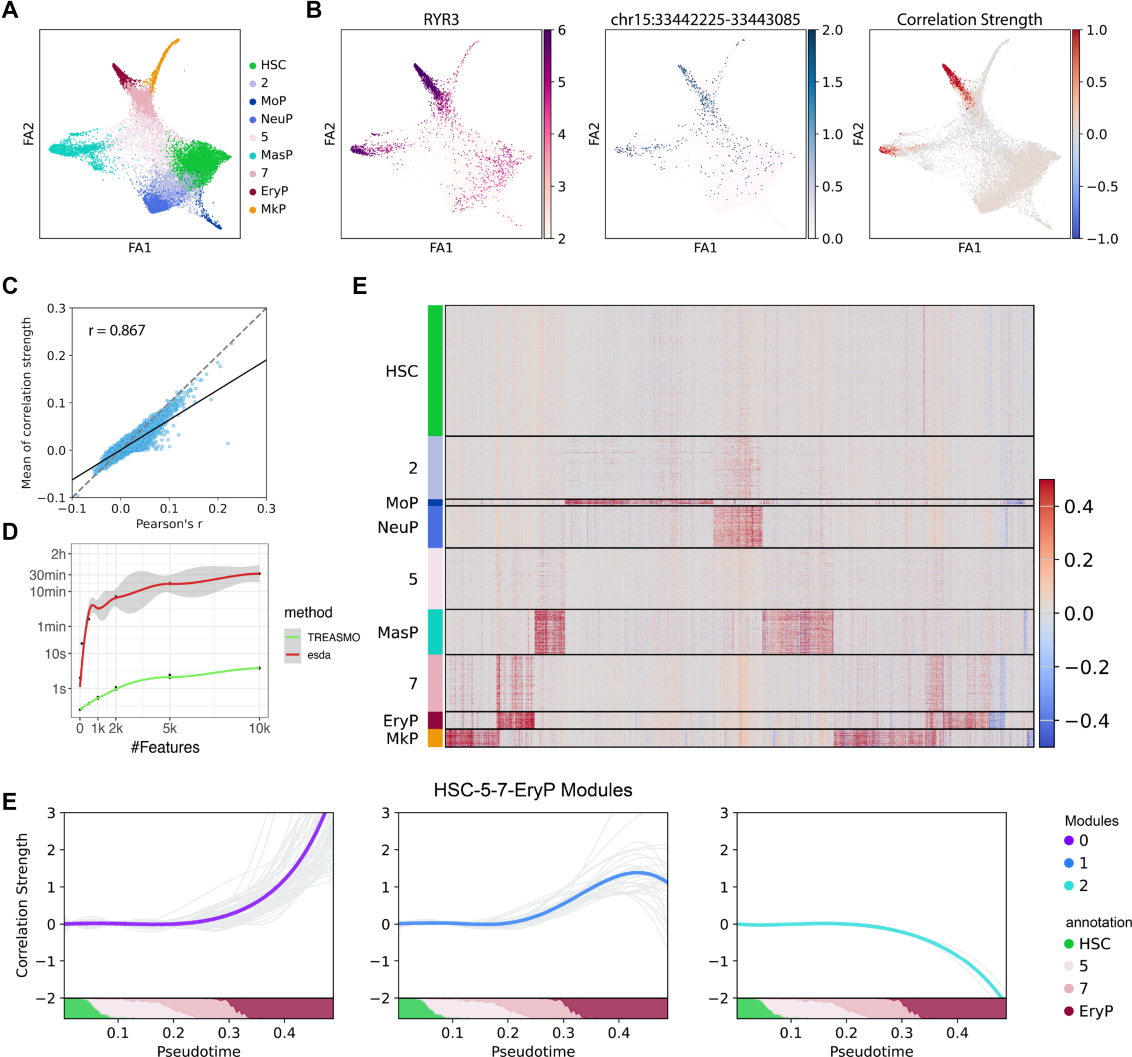
TREASMO showcase on the 10X Multiome HSPCs dataset. (**A**) Dataset visualization on the graph embedding colored by cell types in HSPC lineage commitment. (**B**) Visualization of RYR3 expression (left), openness of its nearby regulatory element (middle) and the gene–peak pair correlation strength (right) inferred by TREASMO. (**C**) Scatter plot shows the consistency between averaged single-cell correlation strength and Pearson's *r*. (**D**) Computational time comparison between TREASMO and esda. Under the limitation of 16 Gb memory and eight threads, the time consumed to calculate the correlation strength index was estimated with different feature numbers. (**E**) Correlation strength heatmap of regulatory markers. Rows are cells, columns are gene–peak pairs and color indicates the correlation strength between each gene–peak pair. (**F**) Dynamic regulatory gene–peak pairs clustered by temporal patterns using a self-organizing map (SOM). Gray lines are gene–peak regulatory curves, and colored lines are module representations. The bottom bar shows the proportion of cell types in each time bin.

In the 10X Multiome HSPCs use case (Figure 2A), we computed the correlation strength for 29 138 gene–peak pairs across all 14 927 cells, storing the output matrix in *MuData.uns[‘Local_L’]*. In Figure 2B, we present a visualization of an illustrative gene and peak correlation example using the *pl* module. Remarkably, the inferred correlation strength (right) exhibits consistency with gene expression (left) and chromatin accessibility (middle), highlighting the reliability of our approach. To systematically validate the strength index, we assessed its consistency with Pearson's correlation. By averaging the individual strength index across the entire dataset, we observed a strong correlation between the resulting global index and Pearson's *r* computed for all 29 138 gene–peak pairs (Figure 2C). This robust alignment substantiates the reliability of the index as an ideal representation of the correlation strength in individual single cells. Furthermore, TREASMO exhibits a substantial improvement in index computation speed compared with the original package developed for geographical studies (10) (Figure 2D).

Based on the correlation matrix, the *ds* module in TREASMO offers a suite of downstream analyses designed to detect regulation changes. Unlike conventional methods that compare Pearson's *r* between two clusters, our approach involves conducting statistical tests by comparing the single-cell level correlation indices of cells in the two clusters, resulting in a more systematic and reliable analysis. This advancement enables the development of general functions for identifying unique gene–peak regulatory markers in individual clusters or between different conditions. In the HSPCs dataset, we utilized the *FindAllMarkers* function and successfully identified regulatory markers for each differentiation stage. The results were effectively summarized and visualized using the volcano plot (Supplementary Figure S2A) and heatmap (Figure 2E) through the *pl* modules. As a validation, we performed Gene Ontology (GO) and motif enrichment analysis with the gene–peak regulatory pairs identified through this process (Supplementary Methods, Supplementary Figure S2). The genes found to be differentially regulated within each progenitor cell group exhibited distinct biological and molecular functions, aligning well with the known roles of these blood cell types (Supplementary Figure S2B). For example, in the megakaryocyte progenitor (MkP) cell population, we observed active regulation and transcription of genes associated with platelet functions (11). Likewise, our motif enrichment analysis unveiled the presence of distinct transcription factors responsible for each lineage differentiation (Supplementary Figure S2C). In the case of neutrophil progenitor (NeuP) cell differentiation, we observed enrichment of known transcription factors such as CEBPD and CEBPA, which is consistent with prior literature (12,13).

The regulation dynamics encompass a more intricate process than mere comparisons between groups of cells. It represents a continuous and ever-changing phenomenon, particularly in cases of HSPCs, where differentiation is continuous (12), and shifts can occur at any time point. To address this complexity, the *ds* module in TREASMO incorporates specialized functions to detect regulation dynamics along a trajectory. In this approach, TREASMO creates time bins, averages the correlation strength within these bins and identifies highly variable gene–peak pairs along the user-defined trajectory path (see Supplementary Methods for details). For instance, along the erythrocyte progenitor lineage (‘HSC - 5 - 7 - EryP’), we successfully detected 98 dynamic gene–peak pairs (Supplementary Figure S2D). In contrast, using only Pearson's *r* to detect gene–peak regulatory pairs resulted in only 59 pairs, even when applying a minimal threshold (|Pearson's *r*| ≥ 0.1, Supplementary Figure S4A). To demonstrate this process, we selected RYR3 and its correlated peaks for further analysis. Leveraging the *pl* module functions, we modeled and visualized the regulation dynamic curves, together with cell type proportion changes, along the trajectory pseudo-timeline (Supplementary Figure S2E).

Motivated by these temporal pattern differences, we developed the ds.*DynamicModule* function. This function clusters all dynamic curves according to their temporal pattern by an SOM (14,15) (see Supplementary Methods). In the case of the ‘HSC - 5 - 7 - EryP’ trajectory, we identified three distinct temporal patterns of regulatory dynamics (Figure 2F). Module 0 exhibits an ever-increasing regulation activity along the trajectory, module 1 shows an increase in regulatory strength at the mid-point but a subsequent decrease, and module 2 reveals the suppression of target gene expression by opening regions. Notably, each temporal pattern corresponds to a unique biological function (Supplementary Figure S3A, B). For instance, module 0′s GO enrichment analysis points to the regulation of hemoglobin-related functions (16), while module 1 is associated with the regulation of cell morphology development functions such as actin filament formation (17). In essence, during erythropoiesis, progenitor cells first undergo morphological changes, followed by a continuous progression towards attaining primary red blood cell functions. In contrast, with the 59 pairs discovered by Pearson's correlation (baseline method), hemoglobin-related functions were totally missed (Supplementary Figure S4B). These sophisticated analyses offered by TREASMO empower researchers to precisely uncover regulation changes, pinpointing specific cell subpopulations of significant interest.

## Discussion

Taken together, our study demonstrates that TREASMO serves as a versatile and powerful tool for novel single-cell multi-omics analysis and visualization, facilitated by its unique single-cell level correlation strength index. We would like to highlight that this index indicates the correlation between genes and peaks in each cell, which may lead to positive values even in cases where genes are less expressed, and their corresponding peaks are less accessible. Additionally, the index reflects each cell's contribution to the overall correlation at the population level and, although it may not strictly range within –1 to 1 in extreme cases, we can still interpret the correlation strength, much like we do with Pearson's correlation. To ensure accurate visualization, we recommend constraining the color range from –1 to 1. Furthermore, as the index follows Mantel's general cross-product association measure (18), it is influenced by dropouts in single-cell data. After scaling the data with zero means and unit variances, dropout values will turn to negatives from zero. These negative but meaningless values will somewhat bias the final correlation results. To mitigate this challenge, TREASMO addresses feature sparsity and dropouts with special attention. We adopt feature sparsity as a quality control metric and incorporate an option that allows zeroing the index when derived from features with dropout values. Nevertheless, future advancements in measuring multi-omics regulatory relationships may explore approaches to mitigate the impact of dropouts altogether.

TREASMO emerges as a robust software solution, empowering researchers to quantify and visualize transcription regulation dynamics within single-cell multi-omics data. We have validated the correlation strength index, and explored its the potential to discover regulatory markers between groups and along the trajectory. With its array of user-friendly and rapid functions and advanced visualization capabilities, TREASMO has the potential to uncover novel insights and unlock previously inaccessible details in single-cell multi-omics datasets, ultimately advancing our understanding of cellular heterogeneity, differentiation and regulatory dynamics. We have made all relevant information available for the reproduction of our presented results in the source code repository (https://github.com/ChaozhongLiu/TREASMO), offering detailed tutorials on installation, usage and function specifics. We will continue to develop TREASMO to meet the ever-evolving needs of the scientific community in single-cell multi-omics analysis.

## Supplementary Material

lqae007_Supplemental_File

## Data Availability

The 10X Multiome HSPCs dataset is available at https://www.kaggle.com/competitions/open-problems-multimodal/data. We downloaded the position frequency matrices (PFMs) of hematopoietic stem cell lineage commitment transcription factors from JASPAR (https://jaspar.genereg.net/) and converted the PFMs into Homer motif files by TREASMO. Motif files are available in the TREASMO GitHub repository https://github.com/ChaozhongLiu/TREASMO. TREASMO is publicly available in the Zenodo repository 10.5281/zenodo.10514626, together with all raw results in the manuscript.
