# Supplementary material for: Quantification and visualization of cis-regulatory dynamics in single-cell multi-omics data with TREASMO
Source: NAR Genom Bioinform. 2024 Feb 2;6(1):lqae007. doi: 10.1093/nargab/lqae007 (PMC10836941; doi:10.1093/nargab/lqae007)
Supplement: lqae007_Supplemental_File [file lqae007_supplemental_file.pdf]

Supplementary Methods

Permutation-based p-value calculation of the global correlation

The significance test for the global correlation is conducted using a permutation approach. To establish a reference distribution of the L index, the paired vectors are shuffled together iteratively, a total of n times. Subsequently, the z-score of the L index under this reference distribution is computed and used as the simulated p-value (see pseudo-code below). Furthermore, the obtained p-values can be adjusted for multiple tests to control for false positives by Bonferroni correction or False Discovery Rate (FDR).

Algorithm Significance test for global L index

1: **for** $x \in \mathbb{R}^n, y \in \mathbb{R}^n$

2: L_{ref} = [ ] (Reference distribution of $L_{x,y}$)

3: **repeat** n times

4: x', y' = **shuffle** 10% x, y together

5: **append** $L_{x'y'}$ **to** L_{ref}

6: **return** $p = \frac{L_{x,y} - \bar{L}_{ref}}{std(L_{ref})}$

Estimation of computation time

We estimated the time consumption for calculating the correlation strength index. To make a fair comparison between TREASMO and *esda*, we limited the computational capacity to 8 threads and 16 Gb of memory. This is done by setting the number of threads with os.environ ['OPENBLAS_NUM_THREADS'] and set the max_RAM parameter of the TREASMO function to 16. No memory limitation was set for esda since it never used more than 16 Gb memory during the experiment.

To test how time consumption changes along with the number of features, we used samples from the 10X Multiome datasets with 5000 cells and selected feature numbers to be 1, 10, 100, 500, 1000, 2000, 5000, and 10000. Time was estimated with the time package in Python.

Compare correlation strength between groups

With the group annotation labels, users can find the differentially correlated gene-peak pairs in each cell cluster or compare conditions by simply calling the function *ds.FindAllMarkers*, *ds.FindPathMarkers*, or *ds.FindMarkers*. These functions execute t-test comparisons, summarizing all results, and providing users with a data frame containing mean correlations within each group, significance test results, and feature sparsity. To streamline the marker selection process, the function *ds.MarkerFilter* allows users to filter pairs based on feature sparsity, mean correlation differences, and p-values. Additionally, setting the plot parameter to True generates a

volcano plot, offering a visual representation of the filtered data frame alongside the selected gene-peak pairs.

Detect regulation dynamics along the trajectory

The method *ds.FindPathDynamics* in TREASMO is designed to select highly variable gene-peak correlation pairs along a user-defined trajectory path using a list of ordered cluster names. The function begins by binning all cells based on the trajectory pseudo-time and computing the average correlation strength matrix, resulting in $\mathbf{L} \in \mathbb{R}^{b \times p}$, where b is the number of user-defined bins. Subsequently, empty bins and bins with values exceeding 2 standard deviations are filtered out. Next, the function calculates the correlation variance and value range for the b gene-peak pairs. By specifying variance and range cutoffs, dynamic regulation pairs along the trajectory are identified and returned as a data frame. Setting the plot parameter to True enables the generation of a volcano plot, providing an optional visual representation of the selected gene-peak pairs.

Modeling regulation dynamics curve

To analyze the dynamics of a specific gene of interest, TREASMO offers two complementary functions: *ds.PathDynamics* for quantification and *pl.PathDynamics* for visualization. Similar to *ds.FindPathDynamics*, *ds.PathDynamics* generates time bin correlation data for the specified gene and its corresponding peaks. Additionally, it summarizes the cell type proportions in each bin along the trajectory. Next, *pl.PathDynamics* leverages Gaussian Process based regression, implemented in scikit-learn (v1.3.0), to fit the time bin data with a continuous curve. The visualization includes a bar representing cell type proportions beneath the curve, providing an intuitive understanding of how different cis-regulatory elements dynamically regulate gene expression along the trajectory.

Functional enrichment analysis

Gene Ontology enrichment analysis and visualization were done using the *clusterProfiler* and *ggplot2* library in R.
Motif enrichment analysis was done using Homer on customized motifs. We downloaded from JASPAR the position frequency matrices (PFMs) of transcription factors involved in hematopoietic stem cell lineage commitment. PFMs were then converted to homer motif files using treasmo.tl.*PFM2Motif*. Homer findMotifGenome.pl was called to compute the motif scores. We set the score 5.0 as the cutoff for a loose matching and calculated the matched peaks ratio with specific motifs in each progenitor cell type. The visualization was done with seaborn *clustermap* in Python. All related results were provided in supplementary figures.

Regulatory Module discovery

The self-organizing map (SOM) algorithm is an unsupervised artificial neural network that groups all observations in a low-dimensional representation while preserving the topological structure of the data. Here, we take gene-peak correlation strength in each time bin as observations and clustered all gene-peak curves by SOM implemented in MiniSOM Python package. Users can run the function multiple times to have the optimized results by changing the SOM shape (som_*shape*, how many modules are needed and what is the similarity among all modules), learning rate (*learning_rate*), number of iterations (*num_iteration*), and regulation power sigma (*sigma*). While

optimizing the SOM results, users can visualize the results by calling the *pl.DynamicModule* function to intuitively understand the results.

Correlation strength heatmap

The function in the *pl* module responsible for plotting correlation strength heatmaps leverages the well-packaged Scanpy function. It creates a new AnnData object with the correlation strength matrix, which is then used to generate the heatmap. However, the standard Scanpy *pl.heatmap* function does not support features clustering. To address this limitation, we implemented Agglomerative Clustering using scikit-learn. We extract the feature orders based on the clustering results and utilize this order to plot the heatmap, enabling effective visualization of the data.

Rapid preparation of gene-peak pairs

TREASMO allows users to compute the single-cell correlation strength for a list of gene-peak pairs. To facilitate the efficient preparation of such pairs, the *tl* module offers two functions: (1) *tl.peaks_within_distance*, which links genes with peaks located within a user-defined genome range; and (2) *tl.TFBS_match*, which links transcription factors with peaks situated in the reference binding site ranges. Both functions employ Pandas-embedded parallel programming to optimize speed.

These functions determine whether the mid-point of peaks falls within the defined range and provide informative data, including the distance to the transcription starting site, distance to the transcription ending site, whether the peak is in the promoter region, whether it resides in the gene body, and the number of base-pair overlaps. By default, links between genes and peaks located in another gene body are removed. However, users can choose to retain these links by setting the *no_intersect* parameter to False.

Color selection

TREASMO adopts a uniform approach to color selection in all types of plots, mirroring the logic employed by Scanpy. The functions initially seek a user-provided color list. If none is provided, they search for keys in MuData.uns that contain the observation column name, such as "ANNOTATION_colors". In the event of not finding a suitable color list, the default rainbow color palette is employed, and colors are automatically selected for the plot. This consistent color selection logic ensures that the plots in TREASMO maintain a coherent and visually appealing representation of the data.

Supplementary Figures

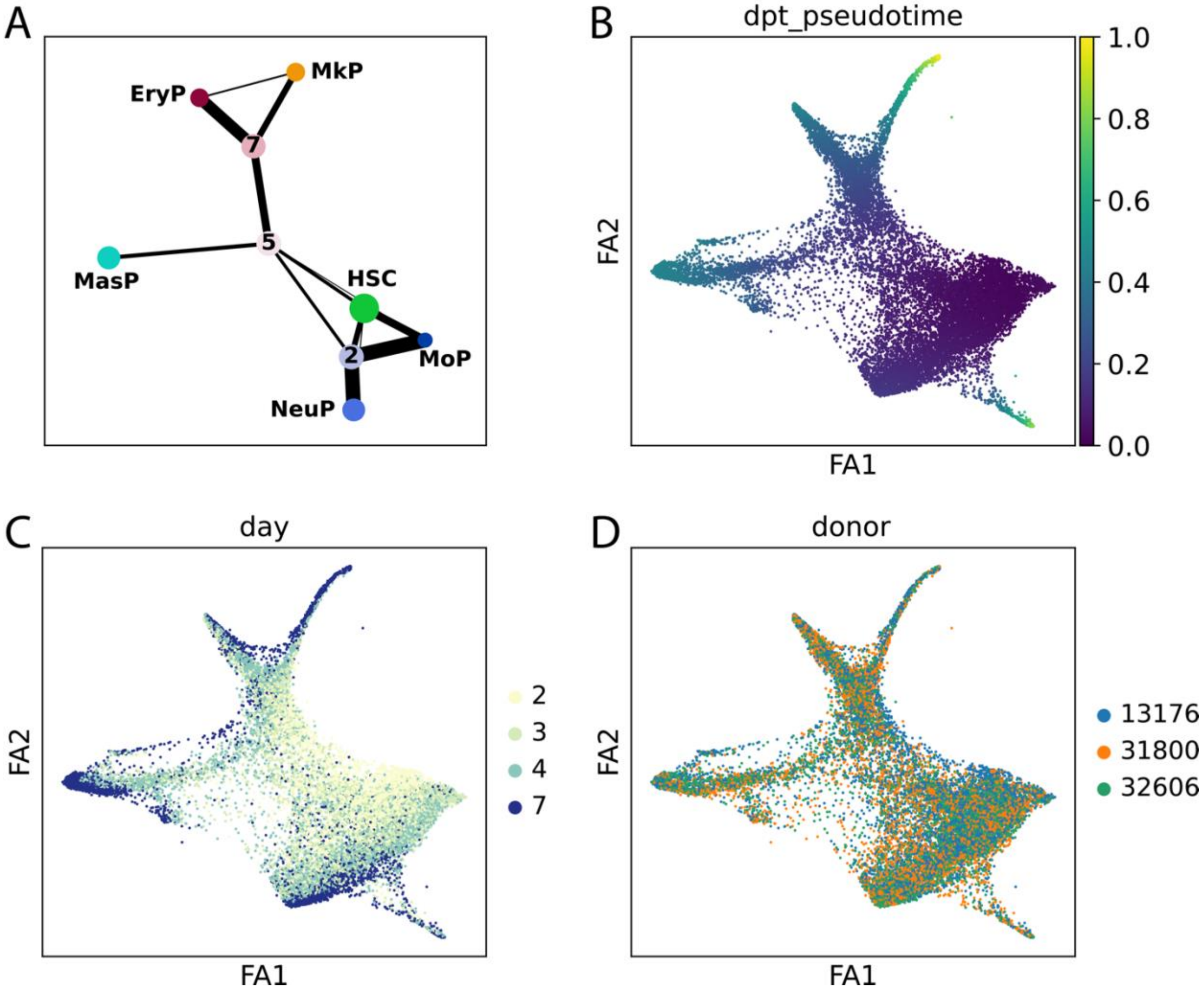

Fig. S1. Overview of the 10X Multiome HSPCs dataset. **A** PAGA graph of HSPC lineage commitment. Then we generated the cell embedding by force-directed graph drawing with PAGA as initialization, labeled cells by **B** pseudotime derived from geodesic distance, **C** days that cells have been plated for before collecting for sequencing, and **D** donor of the cells.

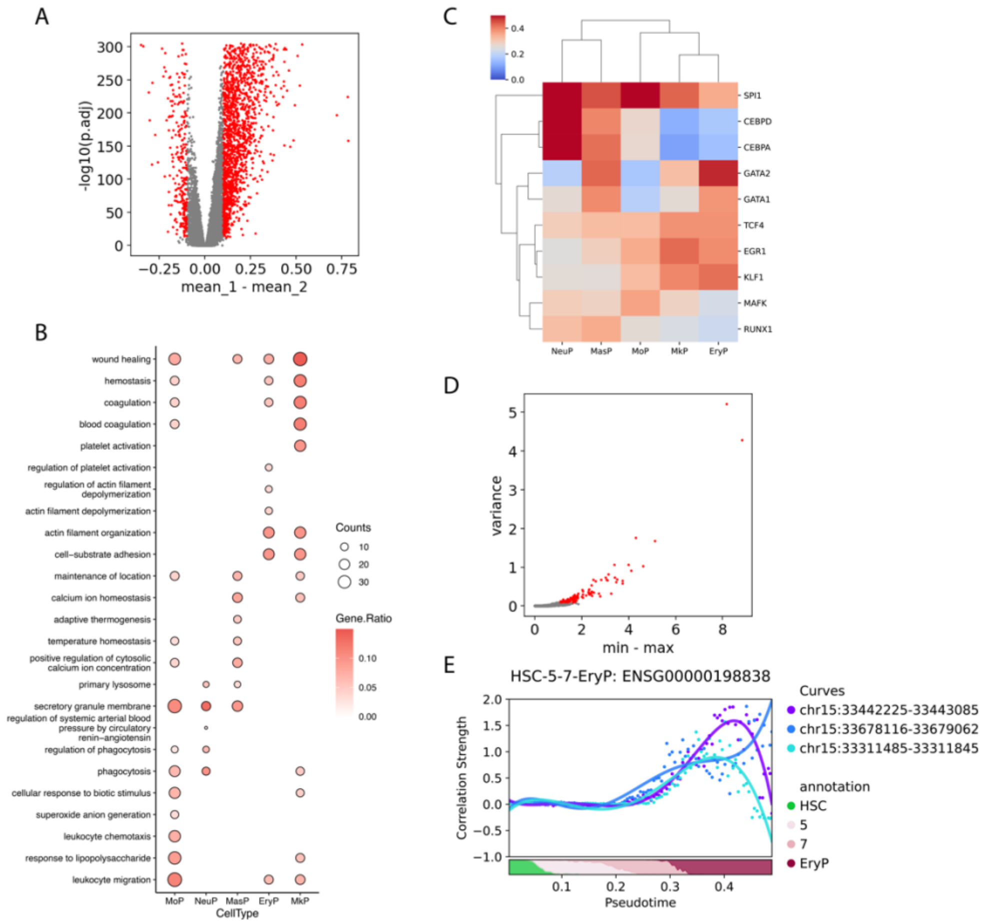

Fig. S2. Intermediate results from TREASMO analysis. **A** volcano plot of HSPCs gene-peak correlation marker discovery results. With the cutoff of at least 0.1 group mean difference, less than 1e-12 adjusted p-value, and feature sparsity less than 10% for gene expression, 5% for peaks, selected gene-peak pairs are colored in red. x axis is the mean difference between group of interest and all others; y-axis is the -log10 adjusted p-value. **B** Gene Ontology enrichment analysis results using genes of regulatory markers in each cluster. **C** Heatmap summary of motif enrichment results using peaks of regulatory markers in each cluster. Color represents the ratio of peaks matched the TF motif. **D** volcano plot of dynamic regulatory pair discovery results in erythrocyte progenitor cell lineage. With the cutoff of at least 0.5 correlation range, and larger than 0.1 strength variance, selected gene-peak pairs are colored in red. x axis is the difference between maximum and minimum correlation strength; y-axis is the variance of correlation in all the time bins. **E** Examples of regulatory dynamics in RYR3 transcriptional regulation.

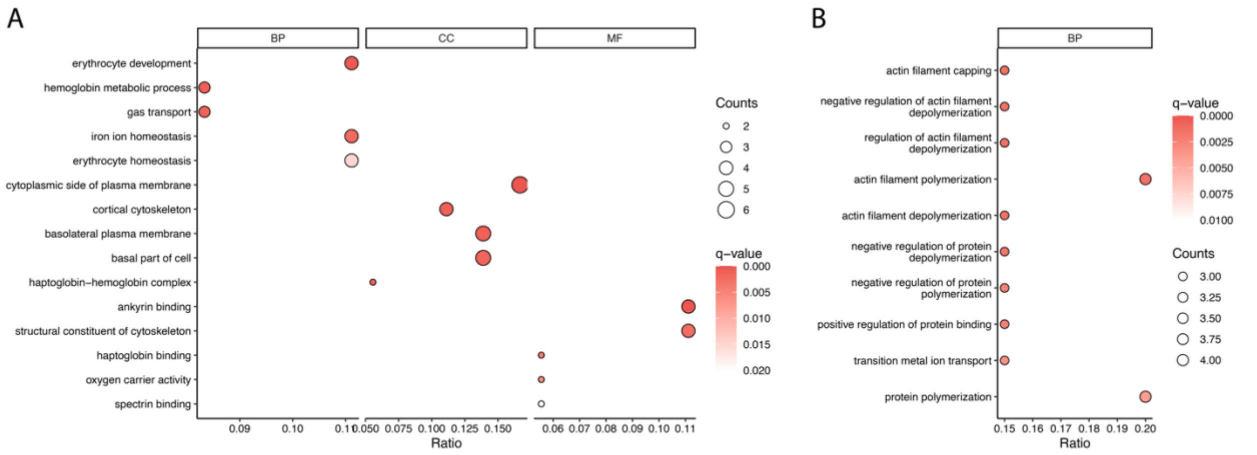

Fig. S3. Functional analysis on discovered dynamic regulatory pairs along erythrocyte progenitor cell lineage. **A** Gene Ontology enrichment on genes from dynamic regulatory pairs in module 0. **B** Gene Ontology enrichment on genes from dynamic regulatory pairs in module 1.

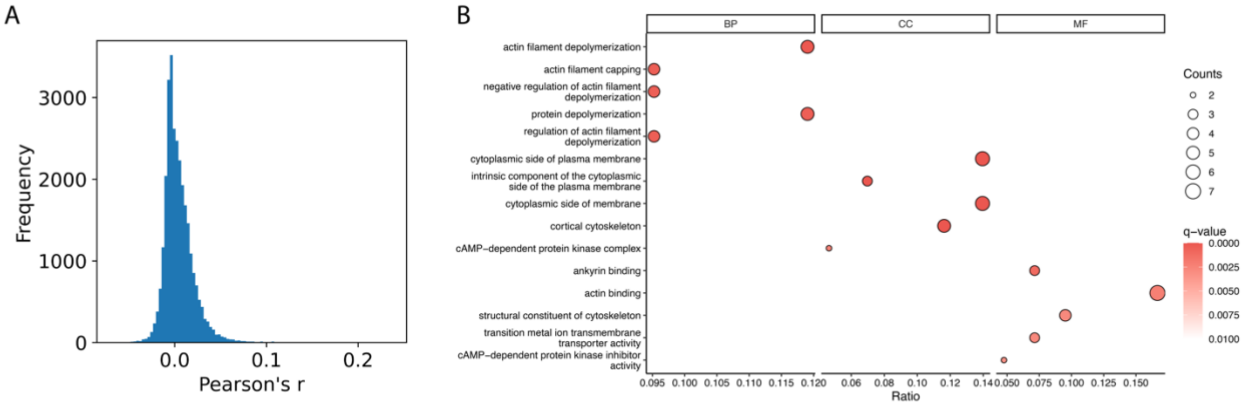

Fig. S4. Detecting important gene-peak pairs with Pearson's correlation as the baseline method. **A** Histogram shows the Pearson's r distribution of all gene-peak pairs. Majority of the values are very low, making Pearson's r unsuitable for this task. **B** Gene Ontology Enrichment analysis results from 59 pairs detected from Pearson's correlation. Gas transport and hemoglobin functions were missed with this baseline method.

TREASMO Function Summary

tl (tools) module	
**Function**	**Description**
feature_sparsity	Add feature sparsity information in MuData.var
peaks_within_distance	Annotate genes with nearby peaks by coordinates
TFBS_match	Annotate TF with binding site regions in the data
PFM2Motif	Convert PFM (Position Frequency Matrix) into Homer Motif file
peak2HomerInput	Convert peak list to Homer accepted input format
run_HOMER_motif	Function to run Homer from Python script. It will prepare Homer required input file and output results in the directory specified
motif_summary	Extract related peaks for motifs of interests from Homer results

core module	
**Function**	**Description**
Morans_I	Calculate Moran's I for all the features in multiome data
Global_L	Calculate the global L index (mean of correlation strength index) for all the pairs
Local_L	Calculate the single-cell gene-peak correlation strength index for all the pairs
Pearsonr	Calculate the Pearson correlation between genes and peaks

***ds* (downstream analysis) module**	
**Function**	**Description**
FindAllMarkers	Discover regulatory gene-peak markers in all groups/clusters
FindMarkers	Compare regulatory gene-peak pairs between two group by t-test
MarkerFilter	Filter markers from statistical test results and return volcano plot
FindPathMarkers	One-to-one comparison of gene-peak correlation among groups in the trajectory path by t-test
TimeBinData	Helper function to generate binned gene-peak correlation data along trajectory
TimeBinProportion	Helper function to calculate binned cell type proportion along trajectory
FindPathDynamics	Detect highly variable gene-peak pairs along the trajectory, return volcano plot
PathDynamics	Quantify regulatory dynamics along the trajectory for a single gene and its regulatory elements
DynamicModule	Cluster gene-peak modules by Self-Organizing Map along the trajectory

***pl* (plotting) module**	
**Function**	**Description**
LocalCor_Heatmap	Cluster gene-peak pairs and visualize the correlation strength matrix by heatmap
visualize_marker	Visualize the gene-peak pair correlation in user provided embedding space
PathDynamics	Complex plot to visualize the gene-peak pair correlation changes along pseudotime + cell type proportion bar

DynamicModule	Complex plot to visualize the gene-peak modules found in the trajectory
DynamicSumMtx	Visualize regulatory element relationships in heatmap by Spearman correlation
